# Applicability of predictive equations for resting energy expenditure in obese patients with obstructive sleep apnea

**DOI:** 10.1590/2359-3997000000228

**Published:** 2016-11-07

**Authors:** Mariana Pantaleão del Re, Camila Maria de Melo, Marcus Vinicius dos Santos, Sergio Tufik, Marco Túlio de Mello

**Affiliations:** 1 Universidade Federal de São Paulo São Paulo SP Brasil Universidade Federal de São Paulo (Unifesp), Psicobiologia, São Paulo, SP, Brasil; 2 Universidade Federal de São Paulo São Paulo SP Brasil Universidade Federal de São Paulo (Unifesp), Biociências, São Paulo, SP, Brasil; 3 Faculdade de Educação Física, Fisioterapia e Terapia Ocupacional Universidade Federal de Minas Gerais Belo Horizonte MG Brasil Faculdade de Educação Física, Fisioterapia e Terapia Ocupacional, Universidade Federal de Minas Gerais (UFMG), Belo Horizonte, MG, Brasil

**Keywords:** Obesity, obstructive sleep apnea, energy expenditure, resting energy expenditure, predictive equations

## Abstract

**Objective:**

To investigate the applicability of predictive equations for resting energy expenditure (REE) in obese individuals with obstructive sleep apnea (OSA) and the effects of OSA severity on REE.

**Materials and methods:**

Twenty-nine obese men, 41.5 ± 7 years old, with moderate and severe OSA were recruited. All subjects were submitted to a clinical polysomnography, body composition, and indirect calorimetry measurements. REE was also predicted by three different equations: Harris and Benedict (1919), Cunningham (1990), and DRI (2002).

**Results:**

No effects of OSA severity on REE were found. The measured REE (2416.0 ± 447.1 kcal/day) and the REE predicted by equations were different from each other ( *F* = 2713.88; *p* < 0.05): Harris and Benedict (2128.0 ± 245.8 kcal/day), Cunningham (1789.1 ± 167.8 kcal/day) and DRI (2011.1 ± 181.4 kcal/day). Pearson correlations showed a moderate positive correlation between the REE measured and predicted by all equations.

**Conclusion:**

Our findings suggest that predictive equations for REE underestimate the energy expenditure in obese patients with sleep apnea. Also, no effects of OSA severity on REE were found.

## INTRODUCTION

Obstructive sleep apnea (OSA) is characterized by repeated episodes of total or partial upper airway obstruction during sleep ( 1 ). A bidirectional relationship between OSA and obesity/weight gain is already described ( 2 ). While OSA can develop as a consequence of obesity, the disease per se might result in energy expenditure changes and excessive daytime sleepiness, leading to weight gain ( 2 , 3 ).

The total energy expenditure (TEE) is determined by different components: resting energy expenditure (REE), thermic effect of food (TEF), and physical activity-related energy expenditure (PAEE) ( 4 ). OSA might modulate energy expenditure in all components of TEE.

Studies suggest that REE is increased in OSA patients. Ucok and cols *.* ( 5 ) showed higher REE in OSA patients compared with a group of snorers, even after corrections for body mass and fat-free mass. Oxyhemoglobin desaturation, successive arousals, and continuous sympathetic activation might be responsible for enhanced metabolism in OSA patients ( 5 , 6 ). OSA also leads to elevated diurnal somnolence and fatigue, which contributes to a sedentary lifestyle and decreased PAEE.

From the clinical practice point of view, it’s important that clinicians accurately estimate energy expenditure in their patients so that higher success rates of nutritional management could be achieved. Many methods for energy expenditure measurements are available nowadays, such as indirect calorimetry, accelerometers, and doubly labeled water ( 4 , 7 ). Indirect calorimetry is a method commonly used by researchers and physicians to measure REE, although it is not available in many hospitals and outpatient health care clinics. In the absence of more accurate methods, many predictive equations were developed during past decades for distinct populations and physiological conditions ( 7 - 9 ). Considering the disturbance in oxygen saturation and energy expenditure found in OSA patients, we tested the hypothesis that predictive equations currently available might not be accurate for this particular population. The aim of the present study was to compare the REE measured by indirect calorimetry with prediction equations in obese OSA patients and, as a secondary aim, to compare the effects of OSA severity on REE.

## MATERIALS AND METHODS

The present study was approved by Ethics Committee of Universidade Federal de São Paulo (#142861) and registered in Clinical Trials (#NCT01985035). Twenty-nine adult obese men (body mass index between [BMI] between 30 and 40 kg/m^2^) between 30 and 50 years old were included in the study. All subjects were submitted to clinical polysomnography for diagnosis and classification of OSA severity. Apneas and hypopneas were scored using standard American Academy of Sleep Medicine guidelines ( 1 ), and apnea-hypopnea index (AHI) was used for OSA severity classification.

REE was measured by indirect calorimetry (Quark CEPET, COSMED) ( 10 ). The measurement was made after an overnight fast. The measurement lasted for 30 minutes with the patients in the supine position and in a temperature- and luminosity-controlled room. Body composition was evaluated by plethysmography (BOD POD) ( 11 ). REE and body composition measurements, and the clinical polysomnography, were all performed with the maximum of one week between procedures.

The measurement of REE by indirect calorimetry was compared with three prediction equations: Harris and Benedict ( 8 ), Cunningham ( 9 ), and the equations recommended by the Food and Nutrition Board (DRI) ( 12 ), as demonstrated in Table 1 .

**Table 1:** Resting energy expenditure predictive equations compared with indirect calorimetry

Author (Year)	Equation
Harris e Benedict (8)	REE = 66,5 + (13,8 x Weight (kg)) + (5 x Height (m)) + (6,8 x Age)
Cunningham (9)	REE = 370 + (21,6 x Fat Free Mass (kg))
DRI (12)	REE = 293 - 3,8 x Age + 456,4 x Height (m) + 10,12 x Weight (kg)

Statistical analysis was performed using SPSS software (IBM Corp., Armonk, NY-version 19.0). Shapiro-Wilk test was used for normality. To observe the effect of OSA severity on REE subjects, the subjects were distributed into three groups based on OSA severity: AHI > 15 ≤ 30 events/h (Group 1), AHI > 30 ≤ 50 events/h (Group 2) and AHI > 50 events/h (Group 3). For comparison between measured REE and REE predicted by equations, and for group comparisons, ANOVA tests were used to make repeated measures. Pearson correlations were also performed between predicted and measured REE values. Bland–Altman agreements plots were made using the MedCalc software (Medcalc Software, Ostend, Belgium) to determine the agreement between measured and predicted REE, and *p* < 0.05 was adopted for statistical significance.

## RESULTS

Twenty-nine adult men, 41.53 ± 7.26 years, obese, and diagnosed with mild or severe OSA were included in this study. Subjects’ characteristics are described in Table 2 . Besides a difference in body mass between Group 2 and Group 3, no other significant differences were found in anthropometric and body composition assessments. As expected, some differences were found in sleep parameters: Group 3 showed higher AHI, respiratory disturbance index (RDI), NREM sleep stage 1 (%), spent more time in SaO_2_ lower than 90%, less NREM sleep stage 3, and less REM sleep than Group 1 and 2.

**Table 2:** Anthropometrics and sleep characteristics of obese patients with sleep apnea

Variables	All participants (Mean ± SD)	Group 1 N = 8 (Mean ± SD)	Group 2 N = 8 (Mean ± SD)	Group 3 N = 13 (Mean ± SD)
Body mass (kg)	106.4 ± 15.2	106.5 ± 11.8	95.5 ± 7.9	113.2 ± 17.1^b^
Height (m)	1.75 ± 0.07	1.76 ± 0.07	1.71 ± 0.06	1.76 ± 0.08
Body mass index (kg/m^2^)	34.7 ± 4.2	34.1 ± 3.8	32.6 ± 2.8	36.5 ± 4.6
Body fat (%)	37.7 ± 5.2	35.2 ± 3.3	37.5 ± 6.0	39.3 ± 5.5
Fat-free mass (%)	62.1 ± 5.5	64.8 ± 3.3	62.5 ± 6.0	60.1 ± 5.8
Fat-free mass (kg)	65.7 ± 7.7	68.9 ± 5.6	59.7 ± 7.1	67.4 ± 7.7
Sleep latency (min)	7.0 ± 7.5	8.1 ± 6.2	5.4 ± 6.1	8.0 ± 9.1
Total sleep time (min)	280.4 ± 158.5	386.6 ± 33.7	343.5 ± 129.9	342.7 ± 53.8
Sleep eficiency (%)	86.4 ± 10.6	89.3 ± 7.1	92.9 ± 3.2	81.2 ± 12.7
NREM Sleep stage 1 (%)	18.9 ± 13.1	11.4 ± 5.9	10.4 ± 4.6	28.6 ± 13.4^a^
NREM Sleep stage 2 (%)	45.6 ± 8.4	46.2 ± 10.2	44.4 ± 6.3	46.5 ± 8.8
NREM Sleep stage 3 (%)	16.9 ± 8.5	22.5 ± 67.0	19.1 ± 7.2	9.1 ± 8.0a
REM Sleep (%)	19.4 ± 6.5	19.9 ± 7.1	26.1 ± 5.2a	15.8 ± 3.0^b^
Total Arousals (n)	101.4 ± 108.2	141.8 ± 56.7	162.7 ± 58.9	345.4 ± 115.7
AHI (events/h)	51.7 ± 24.7	23.6 ± 2.8	40.0 ± 5.9a	76.8 ± 10.4^a,b^
RDI (Events/h)	54.0 ± 22.9	28.9 ± 3.6	42.4 ± 6.4	77.2 ± 10.1^a,b^
SaO_2_ (%)	93.4 ± 1.5	93.5 ± 1.3	93.5 ± 0.6	93.1 ± 1.6^b^
Min SaO_2_ (%)	74.4 ± 8.9	80.2 ± 3.9	77.7 ± 6.0	68.7 ± 9.5^a,b^
TRT SaO_2_ < 90% (%)	18.7 ± 19.1	9.0 ± 9.0	7.4 ± 4.9	32.6 ± 21.3^a,b^

Group 1: AHI > 15 ≤ 30 ev/h; Group 2: AHI > 30 ≤ 50 ev/h; Group 3: AHI > 50 ev/h; ^a^ Different from Group 1 (p < 0.05); ^b^ Different from Group 2 (p < 0.05); Mild OSA: AHI ≥ 15 events/h; Severe OSA: AHI ≥ 30 events/h; NREM: Non-rapid eye movement sleep; REM: Rapid eye movement sleep; SaO_2_: Average Oxyhemoglobin saturation; Min SaO_2_: Minimal Oxyhemoglobin saturation; TRT SaO_2_ < 90%: Total Recording Time with SaO_2_ < 90%.

**Figure 1:**
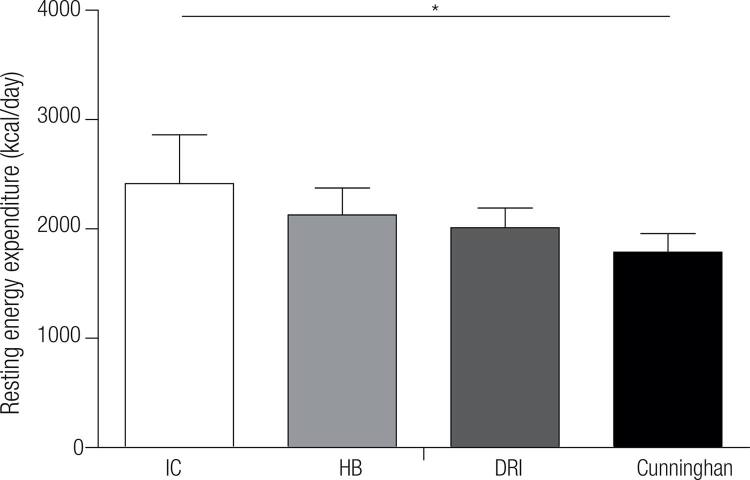
Resting energy expenditure measured by indirect calorimetry and estimated by different equations. São Paulo, 2015. IC: indirect calorimetry; HB: Harris & Benedict equation; DRI: DRI’s equation. Measured repeated ANOVA, F = 49.992 p ≤ 0,05. Values presented in Mean ± Standard Deviation; * p < 0.05.

The measured REE by indirect calorimetry showed higher values of energy expenditure (2416.0 ± 447.1 kcal/day) than that predicted by all equations: Harris and Benedict (2128.0 ± 245.8 kcal/day), Cunningham (1789.1 ± 167.8 kcal/day) and DRI (2011.1 ± 181.4 kcal/day), as demonstrated in Figure 1 .

**Figure 2:**
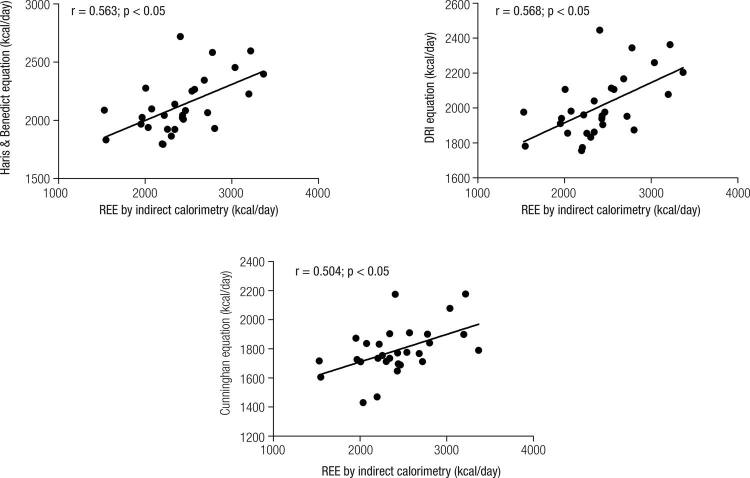
Pearson correlation between measured resting energy expenditure and prediction equations. São Paulo, 2015.

Pearson correlations were made for agreement between different methods and all equations showed moderate correlation with REE measured by indirect calorimetry, as showed in Figure 2 . Between groups divided by AHI, no differences were found in total REE or REE corrected by body mass or fat-free mass ( Table 3 ).

**Table 3:** Resting energy expenditure in all participant and subgroups of AHI. São Paulo, 2015

Characteristics	All participants (Mean ± SD)	Group 1 N = 8 (Mean ± SD)	Group 2 N = 8 (Mean ± SD)	Group 3 N = 13 (Mean ± SD)
REE (kcal/day)	2416.0 ± 447.1	2539.5 ± 400.6	2072.7 ± 424.3	2551.3 ± 399.0
REE/kg (kcal/day)	22.79.8 ± 3.42	23.8 ± 2.4	21.8 ± 4.7	22.7 ± 3.1
REE/kg of FFM (kcal/day)	36.9 ± 6.0	36.9 ± 5.0	35.0 ± 7.5	38.0 ± 5.7

Group 1: AHI > 15 ≤ 30 ev/h; Group 2: AHI > 30 ≤ 50 ev/h; Group 3: AHI > 50 ev/h; REE: resting energy expenditure; FFM: Fat-free mass.

**Figure 3:**
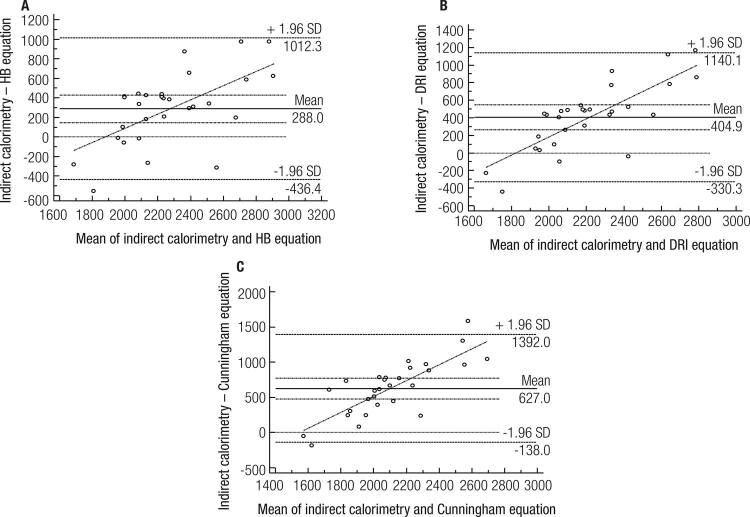
Bland-Altman plots of resting energy expenditure measured by indirect calorimetry and predicted by different equations. São Paulo, 2015. HB: Harris & Benedict equation; DRI: DRI’s equation.

Figure 3 represents the Bland–Altman agreement plots. There were agreements in the measured REE and the values predicted by equations. However, in the resulting the agreements, substantial differences were observed between the methods.

## DISCUSSION

The principal finding of the present study is that the predictive equations underestimate the REE measured by indirect calorimetry in obese individuals with OSA. The relationship between OSA and obesity has been extensively studied in recent years ( 2 , 3 ), and the effects of OSA on energy expenditure have been a target of some studies ( 5 , 6 , 13 ).

In contrast with previous studies that suggested that REE is increased in OSA patients, our study doesn’t confirm any effect of OSA severity on REE. In a pioneer study, Ryan and cols. ( 6 ) compared 14 eutrophic subjects with moderate or severe OSA with 14 healthy control subjects and found increased REE in OSA patients, but failed to find differences when REE values were corrected by fat-free mass values. Kezirian and cols. ( 13 ) observed a positive correlation between REE and AHI in a sample of 212 individuals with OSA. It’s important to note that in these studies, the REE values were not corrected for body mass and body composition. Recently, Fekete and cols. ( 14 ), in a study with 92 individuals with OSA and 19 control subjects, also found a higher REE in patients with OSA. In two other studies, the authors failed to find differences in REE between patients with OSA and healthy subjects ( 15 , 16 ).

The abovementioned studies highlight that OSA has direct effects on energy expenditure. The intermittent hypoxia and sleep fragmentation might lead to a continuous sympathetic activation, increasing energy expenditure during the night ( 17 ). Furthermore, this nocturnal sympathetic hyperactivity could be extended during the day, increasing the REE ( 18 , 19 ).

While REE seems to be enhanced in individuals with OSA, TEE could be compromised. Excessive diurnal somnolence and lower physical activity levels observed in these population might contribute to lowering TEE. Major and cols. ( 17 ) concluded that the more time spent in low saturation rates (below 90%) during the night, the lower the 24 h energy expenditure, which could be a possible explanation for the relationship between OSA and weight gain. In patients with OSA, the nocturnal desaturation and the arousals during the night culminate in higher sympathetic activity and higher energy expenditure. However, in the long term, this mechanism could lead to an adaptive decrease in adrenoceptors activation ( 17 ), resulting in decreased energy expenditure in OSA patients in the long term.

Besides the controversy, the fact is that one way or another, energy expenditure could be affected by OSA, which is an important issue for obesity management in patients with OSA. Due to the limited availability of the indirect calorimetry method in clinical practice, it’s important that physicians recognize these metabolic disruptions and choose the best method to predict energy expenditure of obese patients with OSA ( 19 ). The prediction equations were described as able to provide an easy way for energy expenditure estimation. In our study, three equations were used to compare the measurement of REE: the Harris and Benedict equation, the Cunningham equation, and the DRI equation. The first two were developed using indirect calorimetry data from healthy individuals while the last one used doubly labeled water data from obese individuals ( 8 , 9 , 12 ).

Based on our results from Person correlations and Bland–Altman plots, it’s possible to say that besides the equations underestimating the measured REE, all three equations had at least moderate agreements with the measured values. The Bland–Altman analysis showed some important dispersion of the data. Even though it is the oldest equation, the Harris and Benedict equation showed the biggest agreement with the measured REE. This equation has been considered a reasonable equation for obese individuals ( 20 , 21 ). It’s important to say that it was not developed using data from obese individuals; therefore, some concerns about accuracy in this population persist ( 20 ).

Although the equations underestimate the REE of OSA patients, it should be taken into consideration that the elevation in energy expenditure due to OSA is small compared with the rise in energy intake that a sleep-restricted person might experience, which culminates in an increased predisposition to gain weight.

Based on the data from the present study and the aforementioned literature, we believe that the energy expenditure equations commonly used in clinical practice may have significant biases, considering that OSA can disrupt energy expenditure behavior during sleep, and this change might persist during the day. Clinical physicians and nutritionist should recognize the presence of OSA in obese patients and take into consideration this fact when weight loss is desired.

In conclusion, prediction equations for REE can underestimate the REE measured by indirect calorimetry in obese patients with OSA, despite the agreement between methods and that the severity of OSA had no effect on REE.
